# Unilaterale Netzhautblutungen bei Säuglingen – 2 Fälle von Schütteltrauma?

**DOI:** 10.1007/s00347-020-01038-0

**Published:** 2020-01-29

**Authors:** T. Barth, M. Altmann, C. Batzlsperger, H. Jägle, H. Helbig

**Affiliations:** 1 Klinik und Poliklinik für Augenheilkunde, Universitätsklinikum Regensburg, Franz-Josef-Strauß-Allee 11, 93053 Regensburg, Deutschland; 2 Klinik für Kinder- und Jugendmedizin, Neonatologie, Neuropädiatrie, DONAUISAR Klinikum Deggendorf, Perlasbergerstr. 41, 94469 Deggendorf, Deutschland

**Keywords:** Retinale Blutungen, Schütteltrauma, Kindesmisshandlung, Nicht akzidentelles Schädel-Hirn-Trauma, Subdurales Hämatom, Retinal hemorrhages, Shaken baby syndrome, Child abuse, Non-accidental head injury, Subdural hematoma

## Abstract

Ein 2,5 Monate alter Junge und ein 2 Monate altes Mädchen wurden wegen schwerer Bewusstseinstrübung pädiatrisch behandelt. Bei beiden Kindern fanden sich Subduralhämatome. Bei Verdacht auf nichtakzidentelles Schädel-Hirn-Trauma (NAHI) erfolgte eine Untersuchung des Augenhintergrundes, bei der sich bei beiden Säuglingen unilaterale Netzhautblutungen zeigten. Nach intensiver Differenzialdiagnostik wurde in beiden Fällen der Verdacht auf ein NAHI gestellt und eine rechtsmedizinische Begutachtung initiiert. Wichtig an dieser Fallserie ist, dass die Einseitigkeit von Netzhautblutungen ein NAHI nicht ausschließt.

## Abstract (en)

A 2.5-month-old boy and a 2-month-old girl were admitted to a pediatric intensive care unit with impaired consciousness. Both infants had subdural hemorrhages. Because of presumed non-accidental head injury (NAHI) funduscopy was performed, which revealed unilateral hemorrhage in both children. After intensive differential diagnostics NAHI was suspected in both cases and a forensic medical examination was initiated. This case series is important because it shows that unilateral retinal bleeding does not exclude NAHI.

## Fall 1

### Anamnese

Ein 2,5 Monate alter Junge wurde mit akuter Enzephalopathie und Krampfanfällen auf eine pädiatrische Intensivstation aufgenommen. Die Mutter habe seit dem Vortag ein geschwollenes linkes Auge und seit der Nacht ein Zucken des rechten Beines bemerkt, woraufhin sie den Notarzt verständigt habe. Die von den Eltern erhobene Anamnese bezüglich eines Traumas war leer.

### Befund und Diagnose

**Figure 1:**
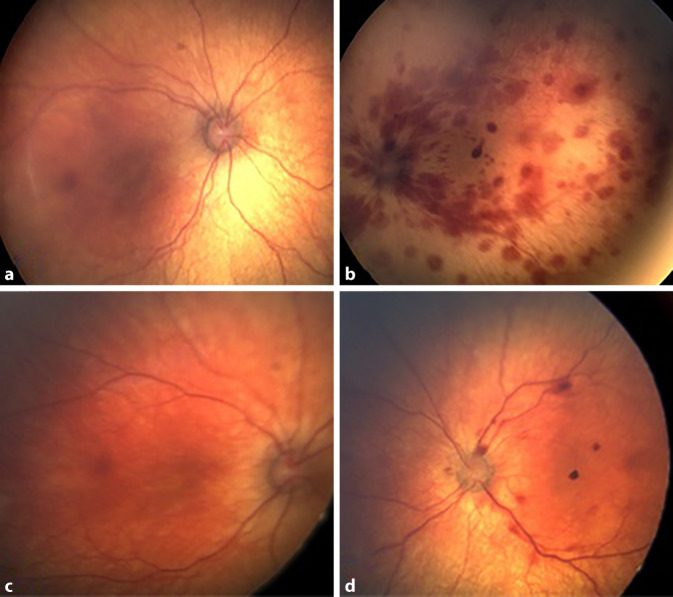

Bereits bei der pädiatrischen Aufnahmeuntersuchung fielen multiple periorbitale Petechien (links > rechts) und ein Hämatom an der linken Wade auf. Des Weiteren bestand ein fokaler Krampfanfall des rechten Oberarms mit Ausweitung auf das rechte Bein. In der Bildgebung des Schädels zeigten sich ein Subduralhämatom links sowie beidseits ausgedehnte zerebrale Diffusionsstörungen passend zu axonalen Schädigungen im Rahmen eines Akzelerationstraumas. Die Skelettstatusuntersuchung nach Leitlinie zeigte keine Frakturen, jedoch eine Pleuraerhebung im Bereich der linken Rippen, die im Verlauf nicht mehr nachweisbar war. Zur Frage der Vereinbarkeit mit einem nichtakzidentellen Schädel-Hirn-Trauma (NAHI) erfolgte eine ophthalmologische Untersuchung. Der Säugling wurde nach medikamentöser Mydriasis mittels digitaler Weitwinkelfundusfotografie (Retcam II, Clarity Medical Systems, Pleasanton CA 94 588, USA) untersucht, und die Fundusbilder wurden telemedizinisch an das ophthalmologische Referenzzentrum übermittelt. Es fanden sich am linken Auge am hinteren Pol und auch weiter peripher Netzhautblutungen unterschiedlicher Ausprägung in verschiedenen Schichten, teilweise mit zentraler Aufhellung. Der Augenhintergrund rechts war unauffällig (Abb. 1a, b). Die umfassende pädiatrische Abklärung ergab keinen Hinweis auf eine systemisch bedingte Ursache der Hämatome wie eine Koagulopathie oder Stoffwechselstörung.

### Klinischer Verlauf

Es erfolgte eine intensivmedizinische Behandlung des Kindes. Unter intensiver antiepileptischer Therapie sistierten die rezidivierenden Krampfanfälle im Verlauf. Bei der augenärztlichen Kontrolle 4 Tage später zeigte sich eine rasche Resorption der retinalen Blutungen (Abb. 1c, d). Nach ausführlicher pädiatrischer Diagnostik und Ausschluss anderer Ursachen für die zerebralen Veränderungen und retinalen Blutungen wurde der Verdacht auf ein NAHI gestellt und eine rechtsmedizinische Begutachtung initiiert. Nach Stabilisierung des Allgemeinzustandes wurde der Junge in eine Pflegefamilie entlassen. Daten zur weiteren funktionellen Entwicklung liegen leider nicht vor.

## Fall 2

### Anamnese

Eine ähnliche Anamnese lag bei einem 2 Monate alten Mädchen vor, dessen Eltern sich mit dem Säugling aufgrund von starkem Weinen, „Verdrehen der Augen“ und Atempausen in der Notaufnahme vorstellten. In der Vorgeschichte hatten die Eltern das Kind bereits im Alter von 1 Monat aufgrund unklarer Hämatome an den Extremitäten in der Notaufnahme vorgestellt, die stationäre Abklärung aber gegen ärztlichen Rat abgelehnt. Das Vorliegen eines Traumas verneinten die Eltern.

### Befund und Diagnose

**Figure 2:**
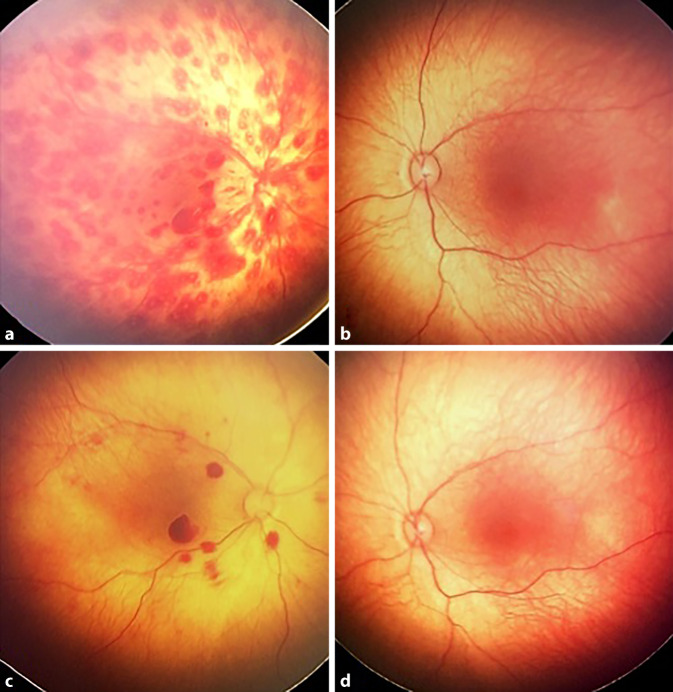

Die pädiatrische Untersuchung ergab ein großes Hämatom an der rechten Flanke sowie eine unklare Unruhe des Kindes mit Bewusstseinstrübung. In der zerebralen Bildgebung zeigten sich beidseits mehrzeitig entstandene Subduralhämatome. Weiterhin erfolgte eine Skelettstatusröntgenuntersuchung, die einen unauffälligen altersentsprechenden Befund ergab. Die ophthalmologische Untersuchung zeigte rechts multiple Netzhautblutungen in verschiedenen Schichten teilweise mit zentraler Aufhellung bei unauffälligem Netzhautbefund links (Abb. 2a, b). Bei unauffälligem Gerinnungslabor und fehlenden Hinweisen auf eine systemische Ursache wurde der dringende Verdacht auf ein NAHI gestellt und das Jugendamt informiert.

### Klinischer Verlauf

Bei rezidivierenden Krampfanfällen erfolgte eine intensivmedizinische Überwachung des Mädchens. Bei der Kontrolluntersuchung des Augenhintergrundes 1 Woche später zeigte sich eine rasche Rückbildung des Befundes mit nur noch vereinzelt darstellbaren Netzhautblutungen rechts (Abb. 2c, d). Die neurologischen Symptome bildeten sich unter antiepileptischer Therapie zurück, und das Kind wurde bei unauffälligem Elektroenzephalogramm in eine Pflegefamilie überführt. Auch hier können leider keine Angaben zur weiteren Entwicklung gemacht werden. Aus pädiatrischer Sicht ist aufgrund der ausgeprägten zerebralen Veränderungen leider mit bleibenden Langzeitschäden zu rechnen.

## Diskussion

Die Diagnose „Schütteltrauma“ hat erhebliche Konsequenzen für das betroffene Kind und die ganze Familie. Die Abgrenzung eines NAHI zu anderen Erkrankungen, die mit retinalen Blutungen einhergehen, ist hierbei nicht immer eindeutig [4]. Während dem Schutz eines potenziell gefährdeten Kindes eine immanente Bedeutung zukommt, sollte gleichzeitig eine falsche Verdächtigung und Stigmatisierung der Eltern vermieden werden [8]. Daher sollte immer eine sorgfältige Diagnostik unter Beachtung anderer möglicher Ursachen der Netzhautblutungen erfolgen [8]. Bei Neugeborenen finden sich recht häufig retinale Blutungen sowohl nach Spontangeburt als auch nach Sectio. Diese Hämorrhagien können in ihrem Aussehen den Blutungen bei NAHI durchaus ähnlich sein, sodass es für den Ophthalmologen insbesondere in den ersten 4 bis 6 Wochen nach der Geburt nicht möglich ist, zwischen dem Geburtstrauma und einem NAHI zu unterscheiden [8]. Neben dem Geburtstrauma selbst kommen bei Neugeborenen und Säuglingen Erkrankungen des blutbildenden Systems (Anämien, Leukämien), Gerinnungsstörungen (Vitamin-K-Mangel), angeborene Stoffwechselstörungen (Glutarazidurie Typ I) oder auch entzündliche Erkrankungen wie Endokarditis, Vaskulitis und Meningitis als Ursache infrage [7].

Ein Schütteltrauma, auch „shaken baby syndrome“ (SBS) genannt, ist charakterisiert durch das Vorliegen einer Trias aus schwerer diffuser traumatischer Hirnschädigung mit akuter Enzephalopathie, subduralen Hämatomen und meist beidseitigen retinalen Blutungen [6, 10]. Am häufigsten sind Kinder im ersten Lebensjahr betroffen, wobei das Risiko für ein NAHI mit 30/100.000 angegeben wird [3]. Die klinischen Befunde sind in ihrer Ausprägung variabel und reichen von Trinkschwäche, Erbrechen und Schläfrigkeit bis hin zu schwerer Bewusstseinsstörung mit Krampfanfällen, Bradykardie und Apnoe des Kindes [1]. Äußerlich zeigen sich meist keine Verletzungen, jedoch finden sich gelegentlich Griffmarken an den Extremitäten oder begleitende Knochen- oder Rippenbrüche [1, 6]. Weiterhin bestehen häufig, wie in unserem Bericht, eine sehr vage Anamnese zum auslösenden Ereignis sowie eine auffällige Sozialstruktur [1]. Die genaue Sozialanamnese (Alter der Eltern, Beruf, soziales Umfeld) konnte retrospektiv in unseren Fällen leider nicht erhoben werden. Als typische Risikofaktoren, die zum SBS führen können, gelten junges Alter der Eltern, niedriger sozioökonomischer Status, Drogen- und Alkoholabusus sowie eine niedrige Frustrationsschwelle und schlechte Impulskontrolle der Eltern [6].

Ätiologisch wird davon ausgegangen, dass es beim Säugling durch repetitive Be- und Entschleunigungen des Kopfes bei gleichzeitig noch schwacher Nackenmuskulatur zum Einreißen der Brückenvenen mit konsekutiver Ausbildung von Subduralhämatomen kommt [7]. Die Enzephalopathie mit Parenchymschädigung des Gehirns entsteht durch den hypoxischen Gefäßschaden und eine traumatische axonale Schädigung [7]. Die retinalen Blutungen sind am ehesten durch Scherkräfte im Bereich des vitreoretinalen Übergangs hervorgerufen, die einerseits eine direkte Gefäßverletzung oder andererseits eine gestörte vaskuläre Autoregulation induzieren [7].

Neben der pädiatrischen körperlichen Untersuchung werden in der Regel eine zerebrale Bildgebung (im Akutfall CT, sonst MRT sowie Schädelsonographie), ein Röntgenskelettscreening, eine Abdomensonographie und umfassende Laboruntersuchungen veranlasst [1]. Der Ophthalmologe wird in der Regel konsiliarisch für die Untersuchung des Augenhintergrundes hinzugezogen. Die Häufigkeit von Netzhautblutungen bei NAHI schwankt je nach Literatur und liegt bei ca. 30–85 % [5]. Die Übersichtsarbeit von Maguire et al. fand eine Häufigkeit retinaler Hämorrhagien von 81 %, wobei die Blutungen in 84 % bilateral auftraten [5]. Wie in den oben dargestellten Fällen (vgl. Abb. 1b und 2a), finden sich bei NAHI häufig zahlreiche Netzhauthämorrhagien in verschiedenen Schichten der Netzhaut verteilt über den gesamten Fundus [7]. Die Blutungen können präretinal, intra- und subretinal lokalisiert sein [3]. Teilweise findet sich wie bei anderen Netzhautblutungen eine zentrale Aufhellung [8]. An Stellen vermehrter Glaskörperadhäsion (Makula, Gefäße, periphere Retina) finden sich die Hämorrhagien gehäuft [8].

Da sich die retinalen Blutungen, wie auch in den von uns beschriebenen Fällen, häufig sehr schnell resorbieren (innerhalb weniger Tage bis 2 Wochen), sollte eine rasche augenärztliche Untersuchung, wenn möglich innerhalb der ersten 24 Stunden, erfolgen [3]. Da es auch nach der Hospitalisierung des Kindes zum Auftreten neuer Blutungen kommen kann, sollten im Verlauf weitere ophthalmologische Kontrollen erfolgen [8]. Neben den Netzhautblutungen werden eine makuläre Retinoschisis oder paramakuläre Netzhautfalten als weitere typische klinische Befunde bei NAHI beschrieben [7]. Prinzipiell können auch Unfälle, z. B. Stürze, zu retinalen Blutungen führen. Der Anteil von Kindern mit Netzhautblutungen nach schweren Schädel-Hirn-Traumata ist jedoch mit 3–5 % sehr gering [3, 6]. Gleichzeitig ist die Anamnese bei unfallbedingtem Schädel-Hirn-Trauma oft eindeutig und mit den vorhandenen Begleitverletzungen vereinbar [3]. Paramakuläre Netzhautfalten bei Kleinkindern wurden bisher nur vereinzelt nach tödlichen Verkehrsunfällen oder Sturz aus sehr großer Höhe beschrieben [3, 8]. Während die Netzhautblutungen und die makuläre Schisis meist zu keiner bleibenden Visusbeeinträchtigung führen, wird die visuelle Entwicklung des Kindes zusätzlich durch die Enzephalopathie und neurologische Auffälligkeiten beeinflusst [8]. Zudem kann eine okulär bedingte Visusminderung durch ein Durchbrechen der Blutung in den Glaskörperraum sowie durch eine Traktionsablatio oder Optikusatrophie hervorgerufen werden [2, 8]. Daher sollten eine augenärztliche Anbindung und regelmäßige Vorsorgeuntersuchung erfolgen, wobei insbesondere bei asymmetrischem Befund das Risiko einer Amblyopie beachtet werden sollte [8].

Obwohl die Bilateralität der retinalen Blutungen in der Literatur häufig als typisches Merkmal angeführt wird [3], gibt es wie in den von uns beobachteten Fällen mittlerweile mehrfach Berichte zu nachgewiesenem NAHI mit streng einseitigem Netzhautbefund [4, 9]. Je nach Quelle liegt die Häufigkeit einseitiger Netzhautblutungen bei NAHI bei 16–26 % [4, 5]. Gleichzeitig wird in der Literatur ein Zusammenhang zwischen dem Ausmaß des Netzhautbefundes und der Schwere des Traumas beschrieben [6]. In unseren Fällen fand sich jeweils ein asymmetrischer ophthalmologischer Befund. Das einseitige Auftreten der Netzhautblutungen könnte mutmaßlich mit einer asymmetrisch erfolgten äußeren Krafteinwirkung auf das Kind zusammenhängen. Hierfür sprechen auch die pädiatrischen klinischen Befunde (Fall 1: Netzhautblutungen links, periorbitale Petechien links > rechts, Subduralhämatom links; Fall 2: Netzhautblutungen rechts, Hämatom der rechten Flanke). Unsere Fallserie soll verdeutlichen, dass die Einseitigkeit des ophthalmologischen Befundes ein NAHI nicht ausschließt. Die Befunderhebung sollte in Verdachtsfällen immer interdisziplinär durch Kooperation von Ophthalmologie, Pädiatrie und Rechtsmedizin erfolgen.

## Fazit für die Praxis

- Retinale Blutungen bei Säuglingen können vielfältige Ursachen haben und müssen umfassend abgeklärt werden.
- Die Trias aus retinalen Blutungen, Subduralhämatomen und Enzephalopathie ist typisch für ein nichtakzidentelles Schädel-Hirn-Trauma (NAHI).
- Aufgrund der schnellen Resorption der Netzhautblutungen sollte eine zügige ophthalmologische Mitbeurteilung erfolgen.
- Eine augenärztliche Anbindung und regelmäßige Vorsorgeuntersuchung sind sinnvoll.
- Die Einseitigkeit retinaler Blutungen bei Säuglingen schließt das Vorliegen eines NAHI nicht aus.
